# Implementation of a Classroom Program of Physiotherapy among Spanish Adolescents with Back Pain: A Collaborative Study

**DOI:** 10.3390/ijerph17134806

**Published:** 2020-07-03

**Authors:** María Blanco-Morales, Vanesa Abuín-Porras, Carlos Romero-Morales, Mónica de la Cueva-Reguera, Blanca De-La-Cruz-Torres, Isabel Rodríguez-Costa

**Affiliations:** 1 Faculty of Sport Sciences, Universidad Europea de Madrid, 28670 Madrid, Spain; vanesa.abuin@universidaderopea.es (V.A.-P.); carlos.romero@universidadeuropea.es (C.R.-M.); monica.delacueva@universidadeuropea.es (M.d.l.C.-R.); 2 Department of Physiotherapy, University of Seville, 41009 Seville, Spain; bcruz@us.es; 3 School of Nursing and Physiotherapy and Podiatry, Alcalá de Henares University, 28805 Madrid, Spain; isabel.rodriguezc@uah.es

**Keywords:** adolescent, back pain, physiotherapy activities

## Abstract

Background: The prevalence of back pain in adolescents is steadily increasing, with negative repercussions on students’ social and academic life. This study sought to improve the ergonomics and musculoskeletal health of adolescents in secondary school by implementing physiotherapy actions within the educational context. Methods: A qualitative collaborative action research approach was used, comprising 49 students, 9 teachers, 11 family members, and 9 physiotherapists. Workshops on ergonomics, stretching, and massage were held. Visual materials were developed to support the assimilation of the information given at the workshops. Data collection included field notes, reflexive diaries, in-depth interviews, and discussion groups. The data were analyzed using the Atlas.ti 6.0 program (Scientific Software Development GmbH, Berlin, Germany). Results: The presence of a physiotherapist in the school context facilitates the acquisition of healthy postural habits. All the adolescents perceived a decrease in back pain after undergoing the program. Conclusions: physiotherapy activities offer students new tools to decrease their back pain and improve their health.

## 1. Introduction

There is growing concern among teachers, health professionals, and parents regarding the increase in back pain among adolescents [1,2]. An investigation recently undertaken in 2019 concludes that chronic back pain is a considerable public health worry [3] which has consequences at both educational and health levels; this has a significant impact on health system costs [4] and consumes considerable healthcare services [5]. A recent study carried out in 2020 finds that back pain during this period of life is a matter of concern since it has health implications in adulthood [5,6]. In the short term, the consequences include an increase in medical care and school absenteeism and a restricted ability to perform daily activities [7], which is correlated with depression among adolescents [8] and can affect their social behavior [9]. Along these lines, a study analyzing the relationship between physical activity and lower-back pain in Danish schoolchildren using retrospective data reported that between 5% and 19% of adolescents suffer from recurrent low back pain [10]. Likewise, other reports show that, in late adolescence, the prevalence of back pain reaches similar levels to adulthood [6,11] and is a predictor of the long-term course of this health challenge [12]. 

According to a retrospective study which analyzed backpack injuries in school children, the government should adopt ergonomic measures and solutions to prevent this growing pathology among secondary school students [4]. Any change or actions proposed should involve their closest environment, such as teachers and family members [11]. These activities can be taught by a physiotherapist, but educators could be included in the teaching process [9].

A cross-sectional study performed in Australia [5], reported that adolescence, which is the period in which secondary education takes place, is an ideal time to establish and instill basic health concepts to favor a teenager’s physical and mental wellbeing, potentially continuing to benefit from this into adulthood. During adolescence, the high level of activity which characterizes infancy decreases, and this inactivity plays a negative role in the appearance of back pain [8]. In addition, according to a review study, prolonged sitting is one of the greatest risk factors of back pain [12]. 

The lack of flexibility, defined as the ability to freely move a joint though its normal range of motion [11], predisposes a person to the appearance of musculoskeletal lesions and functional impairments [12]. Along these lines, a cohort of high school students in Montreal, Canada, was followed prospectively over a 12-month period [13], showing that decreased flexibility in the ischial-peroneal-tibial muscles constitutes a risk factor for the appearance of low back pain [14]. 

One of the tools used in the literature [15] which has shown to be effective for improving flexibility are analytical myotendinous stretches, which aim to increase the flexibility and elasticity of muscles and tendons. Moreover, an observational study conducted in California [16] proposes a protocol of treatment for back pain in childhood and teenage years in which the physiotherapist is included as an essential figure within the process of rehabilitation. One of the techniques commonly used by physiotherapists for the treatment of back pain is massage, considered to be a safe tool, without risks or secondary effects for the treatment of pain of muscle origin [17]. Other authors affirm that the most common treatment for back pain is medication [18]. However, other studies conducted in Germany [19] affirm that the benefits of massage are maintained for longer periods compared with the benefits obtained from conventional treatment with medication. Another aspect collected through literature reviews [20] is that massage represents an alternative to pharmacological treatment, as treatment with medication presents greater secondary effects than massage therapy. The potential decrease in back pain using massage has shown to facilitate the adoption of ergonomically correct postures [21]. In addition, according to a randomized study conducted in Canada [22], massage produces a decreased level of anxiety and stress.

To summarize, studies analyzing the different treatments available for the management of back pain recommend the use of passive therapies as a complement to other active therapies such as stretches or exercise [23]. However, despite the prevalence of back pain in society and the many treatments used for this recurrent problem, few treatments have a solid and conclusive evidence [24]. To date, no studies are available based on research-action methods in which students, teachers, and family members are included together with health professionals (physiotherapists) to design tools for improving back pain among students.

The main aim of the present study was, firstly, to explore the current situation of the academic reality in Spain and the health status of adolescents, with the purpose of improving the adolescent health through the implementation of a physiotherapy program within the school context.

## 2. Materials and Methods

### 2.1. Design

To achieve these objectives, we have designed a qualitative method that allows researchers to perform a first exploration of the current situation of the academic reality in Spain and the health status of adolescents; after this initial analysis, activities were performed to improve the health of the study participants. Particularly, the qualitative methodology used for the development of this project is framed within collaborative action research, where physiotherapy activities are agreed by all participants.

### 2.2. Ethics

The study was approved by the Ethics Committee of the Rey Juan Carlos University, Spain (CIPI 060520165416). The present study was adhered to the ethical standards of the Declaration of Helsinki. In addition, a consent inform form was obtained from all the participants before the beginning of the study.

### 2.3. Participants

Data were gathered at two high schools, a public high school (PBHS) and a private school (PRHS) of the Community of Madrid (Spain). This study used theoretical intentional sampling. The sample was represented by 78 participants: 49 students, 9 teachers, 11 family members, and 9 physiotherapists. All the students had been diagnosed by their family doctor for back pain during the last year. All the students were studying their fourth year of secondary education in the Spanish education system. 

The demographic result showed that the mean age was 15.67 ± 0.71 standard deviation (SD), and the body mass index (BMI) of the participants was 16.70 ± 2.81 SD. The distribution of gender was that 41% of respondents were females and 59% were male. The economic status of the students was that 41% of respondents were medium-high economic level, 37% had a low economic status, and 22% were medium economic level.

### 2.4. Instruments

The data collection tools used were interviews, reflexive diaries, discussion groups, and field notes. Table 1 describes the information on the duration of interviews and discussion groups.

All the tools employed in this research followed a qualitative, descriptive-cross-sectional method via semi structured interviews performed in situ. Firstly, after the literature review a series of questions were drafted based on aspects that could influence a student’s back pain. Subsequently, the research team filtered the questions, representing the three dimensions that the different questions were classified into: characteristics of pain (frequency, area, intensity, duration of symptoms), how the pain affects the students’ day-to-day, the treatments carried out, the previous knowledge of ergonomics or physiotherapy, and action proposals and suggestions. Subsequently, a group of experts was gathered, comprised of nine physiotherapy professionals. The group of experts consisted of two professors from different Spanish universities and seven physiotherapists with a minimum of 10 years of professional experience in their field and whose participation in the study was voluntary and impartial. Prior to the meetings, they were given an interview guide to review. Additionally, they were informed that all the sessions were recorded and handled with complete confidentiality for later analysis. Once the results of the expert group were obtained, the interviews were redesigned and restructured. 

### 2.5. Procedure

The duration of this study was 27 months, beginning in April 2014 and finishing in June 2016, consisting of three general study phases. The first phase was exploratory, based on planning and project design. The second phase was the implementation of the project, and the third phase consisted of data collection, the analysis of information, and stepping back from the scene. 

Prior to the phase of implementation, informed consent was signed by the students as well as their family members or legal guardians, as well as by the family members and teachers who participated in the present study.

The intervention took place between 1 September 2014, and 27 March 2015, for a total of seven months. All the physiotherapy (PT) activities took place in the usual classroom where the students held their classes in order to provide tools for the management of pain within the context of the participants’ daily life. These were performed during school hours using material which formed part of the participants’ daily context.

First, in-depth interviews were conducted individually with each participant (students, teachers, and family members). Thereafter, discussion groups were performed in which the PT activities to be performed were agreed upon. Both the interviews and the discussion groups were audio recorded to facilitate subsequent data collection and analysis. 

The field work included the implementation of 16 PT activities: 3 ergonomics workshops, 3 stretching workshops, 3 five min classroom stretching, 3 posters, 2 massage workshops, 1 project on occupational risk prevention, and 1 video whose protagonist is a superhero who watches over the health care of students. 

The workshops were taught by the main researcher. All the workshops were based on active methodologies, as the intention was for the students to participate in their own learning. All the PT activities, following the procedure of intervention-action qualitative research, were agreed with the students and teachers after analyzing the information from initial interviews and focus groups. The PT activities were carried out in the hours of tutoring or physical activity, always adapting to the availability of teachers depending on the academic program. Each workshop lasted for 1 hour and was performed in the usual classroom where the students’ classes were held. During all the workshops, the external observer was present to keep field notes on the activities performed. The 5 min of stretching was performed three times per week, prior to class. Both the poster as well as the project on occupational risk prevention and the video were designed by the students themselves together with the teachers and the main researcher. The content of the audiovisual material was based on the knowledge learnt during the workshops on stretching and ergonomics. The posters were placed in the classrooms where the student received classes. Lastly, upon completing each PT intervention, the students and teachers wrote in a reflexive diary, for which they were given 10 min.

### 2.6. Statistics Analysis

Once all the data were collected, the interviews were transcribed using Dragon Naturally Speaking v.12 (Nuance Communications®, Massachusetts, USA). Following this, a researcher categorized the interviews using Atlas.Ti v.6.0 (Scientific Software Development GmbH, Berlin, Germany), establishing the most relevant codes and comments. Axial coding was applied to relate the subcategories and codes and to create conceptual families. Finally, concept mapping techniques were employed to show the relations and elements that constitute the phenomena and experiences to be studied in a more graphic and intuitive manner. 

In this study, the techniques of data collection include 5 discussion groups, 119 interviews, 240 diaries, and 16 field notes were conducted. All the instruments used were based on methods of observation and conversation. Prior to the interview and discussion groups, the participants were informed that they would be recorded but that their anonymity would always be respected.

## 3. Results

The characteristics of the sample are summarized in Table 2.

The characteristics of back pain in adolescents are presented in the context of the study. The main cause of back pain was poor ergonomics. The weight of the backpack is the second-leading cause of back pain. Finally, sports-related injury was collected as the third cause of back pain. As far as the frequency of back pain is concerned, 37% of students have pain one or several days a week, while 28% of the interviewees report a daily frequency of back pain. Finally, 7% of interviewees report that the frequency of back pain is once a year. Lastly, it is important to highlight the location of the pain; the lumbar area with 39% of the participants was the most frequent location, followed by 32% who located the pain at the dorsal area and, finally, 29% of students who located it at the level of the trapezes and cervical area. Most of them presented bilateral pain in a single vertebral area.

The results were grouped into three categories or conceptual families, formulated as new knowledge acquired, improvements experienced by the participants, and the daily applicability of the physiotherapy activities. Table 3 describes the results obtained after the analysis of the discussion group recordings; the in-depth interview recordings; the reflexive diaries kept by the students and teachers; and, lastly, the field notes taken by the physiotherapists as external observers. 

**Figure 1:**
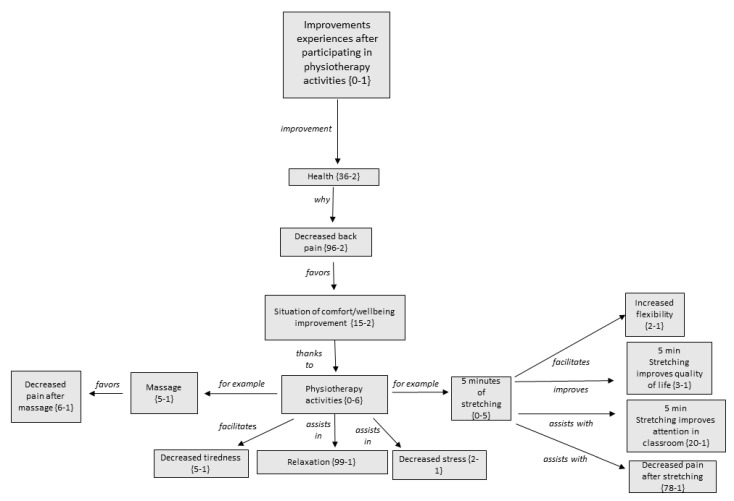
Tree-structure of improvement experiences after participating in Physiotherapy activities: relation between families, codes, and key codes.

Figure 1 gathers the learning of new content acquired after participation in PT activities. The relation between the different codes or subcategories that conform this family or category are gathered (Figure 1). The number in parentheses indicates the number of times that code (comment) has been collected by participants. The number in parentheses indicates the number of times that code (comment) has been collected by participants and the number of times that code is related to similar concepts.

*“It’s really fun, and, also, we have learned new things so that our back doesn’t ache.”*

(FI, PRHS, ST4, 1.11)

It is important to highlight that, as a result of the program, the participants learnt how to identify ergonomically incorrect postures. In addition, the participants’ quotes reaffirm their new understanding regarding postural self-analysis and the improvement of body awareness.

*“I am going to be more careful when doing it, now I think about it, before sitting poorly, I correct myself.”*

(FI, PBHS ST18, 2.11)

The students felt that they had learned many stretches which they had not previously heard of, as many of them commented that they had never been taught stretches in physical education class.

*“I managed to perform the stretches well and the back pain eased a bit.”*

(FI, PBHS ST3, 4.23)

According to the teachers, the information provided by the PT activities was powerful, as the students were able to learn about and acquire tools for improving and preventing their back pain. Beforehand, most students were unaware of how to manage or treat this pain.

*“I believe it is very important, the informative aspect, so that later this can be integrated into their day to day life.”*

(FI, PRHS, T3, 10.37)

The fact that the students knew about the consequences of maintaining poor postures helped support a change in the students’ postural habits, as, when the adolescents saw the consequences associated with poor ergonomics, their level of motivation increased.

*“Now I am more aware of the risks than before.”*

(RD, PRHS, ST9, 46)

**Figure 2:**
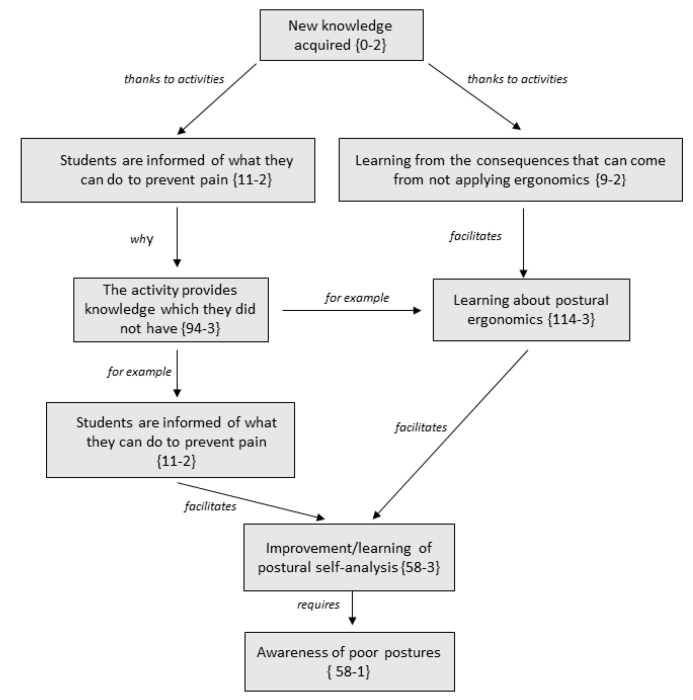
Tree-structure of new knowledge acquired by students: relation between families, codes, and key codes.

Figure 2 displays the tree-structure regarding the improvements experienced after participation in PT activities. The relationship between the different codes and subcategories conforming this family or category is shown. The number in parentheses indicates the number of times that code (comment) has been collected by participants and the number of times that code is related to similar concepts.

Among the wide range of improvements experienced after participation in PT activities, it is important to note that most participants affirmed that their back pain had decreased after participating in the PT activities.

*“The workshops have helped me to change, I have truly felt that they have been useful. In my daily life, this is noticeable and little by little, the back ache that I had starts to go away.”*

(FI, PBHS, ST1, 00.39)

The stretches were also considered to be an effective tool for the management of back pain in teenagers.

*“Sometimes, when I am studying for quite some time, my back or my neck starts aching, sometimes I do one of the stretches that we did… then I notice the muscle is much more relaxed.”*

(FI, PBHS ST10, 00.53)

The participants highlighted that the workshops provided them with a feeling of overall relaxation. They learnt tools to help relax their muscles. Specifically, the workshops on stretching and massage, together with the 5 min stretches that the teacher performed in the classroom, provided relaxation for the back muscles, which allowed the students to better manage activities in their daily life.

*“It is helpful not only to relax muscles, but also physically and mentally, to relax and stretch our legs, it’s interesting.”*

(FI, IBF, ST18, 2.29)

During the interviews, the students described experiencing improved concentration after performing the 5 min stretches in the classroom.

*“The 5 minutes that we do are quite good, it helps prepare us, sometimes before the exams, we do certain postures or we do yoga, it helps a lot, it relaxes us, and we concentrate better during the exam.”*

(FI, PBHS ST2, 2.11)

Lastly, it is important to highlight that during the interviews, some students revealed that they now associated the workshops with an improvement in their state of health and wellbeing.

*“I know how to care for my body a little bit better, if something hurts in one part of the body, now I know how to try and make it be better, how to cure it, all in all, it’s done me good.”*

(FI, PBHS ST4, 7.55)

**Figure 3:**
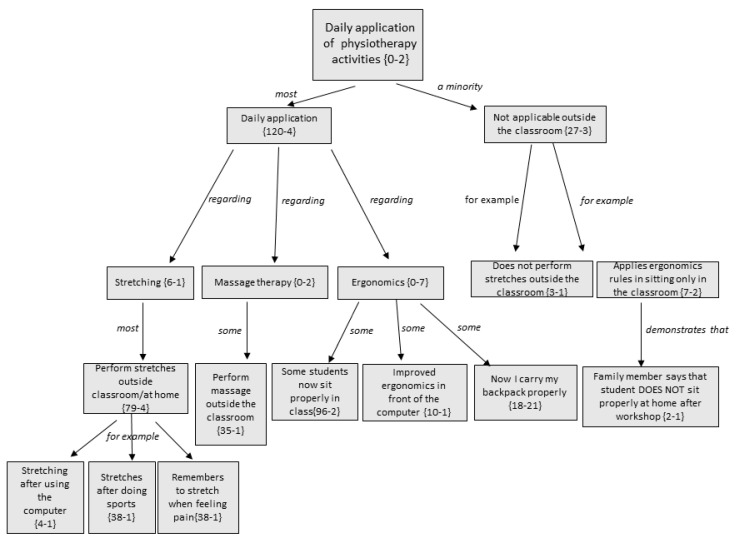
Tree-structure of the daily application of PT activities: relation between families, codes, and key codes.

Figure 3 represents the tree-structure on the daily applicability of PT activities. The relationship between the different codes or subcategories that conform this conceptual family or category can be observed (Figure 3). The number in parentheses indicates the number of times that code (comment) has been collected by participants and the number of times that code is related to similar concepts.

The students commented that the PT activities which they applied the most were those concerning ergonomics and stretching. Perhaps this is because massage is a more complex action for them to perform in their daily life. Instead, they understand massage as being an action to be performed sporadically when their back ache becomes more intense.

*“These are all very important workshops, however, some are more important than others and there are others which you perform all day, such as sitting, because you spend many hours here and the one on the backpack because you carry it almost all day long.”*

(FI, PBHS, ST1, 00.14)

Whatever was learnt was then extrapolated to outside the classroom, beyond the school or high school. This finding was confirmed by both students and family members. Most students used stretches more as a treatment for pain rather than for prevention.

*“Now at my home, I usually stretch on some afternoons when I have time and you can really notice the difference. Then, especially on the following days, when I go back to class, I don’t feel my back is so strained, I think it is quite good.”*

(FI, PBHS, ST8, 00.56)

The improvements experienced by participants are highlighted regarding adopting a correct posture when using the computer.

*“The truth is, now we can say “sit properly", they already know what it means to sit properly, in two words they are able to go back to the position in which they know they should sit; a straight back, a supported back, it’s faster.”*

(DG, T63, PRHS, T1, 4.18)

Many students started to wear their backpack properly after performing the ergonomics workshop. One of the reasons for this change was the information that the students received on the possible future consequences of having a backpack that is poorly positioned. Another reason which lead to this change is that the students felt the difference associated with wearing their backpack positioned correctly vs. incorrectly. Many students referred that, despite carrying the same number of books, when they positioned the backpack correctly, the backpack felt lighter.

*“I have found it very useful because before I would carry my backpack down by my bum and I would struggle, and it was hell and suddenly you said I should raise it higher, and I thought to myself “it doesn’t weigh a thing! I was very surprised.”*

(FI, PBHS, ST5, 1.49)

The findings also revealed that some students did not apply any of the PT activities outside the educational center. Thus, in the classroom, having the posters hanging up was an effective reminder to maintain a good posture; however, at home without a reminder of “correct ergonomics”, they forgot to do so. Others commented that once they get home they relax, which makes them naturally adopt postures used in the past, as the application of ergonomically correct postures entails an effort.

*“At home not so much, maybe here when I am here yes, but there, at home, it’s something else. The posters really help to remember it more”*.

(FI, PRHS, ST7, 1.37)

## 4. Discussion

The PT activities proposed during compulsory secondary education promote the acquisition of knowledge in real contexts, which facilitates the integration of the same and increases student motivation [25]. The use of practical workshops in real contexts promotes the application of teaching and learning methodologies which are more active and which facilitate student learning [26]. The present study took place within the context where the students spend most of their daily life. Additionally, the intervention was designed based on the activities and postures which adolescents typically adopt in their daily life, meaning that the usefulness of the program was prioritized, as highlighted by the participants. 

The lack of knowledge on the anatomy of the spine and the consequences derived from adopting ergonomically incorrect postures daily can be risk factors for back pain [27]. Therefore, health education programs reduce the risk of suffering back pain due to the extensive knowledge on ergonomics and anatomy which these programs provide. In the present study, workshops on ergonomics and stretching were performed. In these, the students learnt the anatomy of the body in a very educational manner, and most students highlighted that these activities had helped improve their knowledge.

Health programs for the back have demonstrated to have benefits for reducing pain intensity, disability, and the frequency of back pain [23,28]. The PT activities obtained benefits which are in line with previous reports [23,28] by decreasing the intensity of back pain, and disability, by allowing teenagers to perform their sports practice without pain and reducing the frequency with which the student suffered pain, at times leading to an overall disappearance of pain.

The performance of health education programs for the back increases body awareness, therefore improving the postural habits of students in general [29]. After the intervention performed in the present study, most of the participants were able to increase their body awareness and experience an improvement in their body awareness.

O’Sullivan et al. (2011) [30] defend that the adoption of ergonomically correct postures can have positive influences on student motivation, writing, and academic results. In the present study, the participants highlighted that the 5 min of stretches performed at the beginning of classes improved their attention and involvement in class. Therefore, perhaps this leads to a higher academic result in the long term. Further studies are required, based on studying the academic benefits provided by PT activities. 

Furthermore, the positioning of a student’s backpack is a determinant for the health of their back [31]. Most adolescents carried their backpack too low, at the level of the lumbar spine, influenced by their social environment. During adolescence, drastic and rapid changes take place in a young person’s physical, mental, emotional, and social development, which leads to ambivalences in the process of seeking a level of balance between oneself and society [32]. The social influences which the students in this study were subjected to decreased after participating in the ergonomics workshop, as the correct placement of the backpack was no longer an isolated action adopted by only one teenager, but rather most of the class had now adopted this change.

Extensive evidence shows that the acquisition of habits and routines favors longevity and wellbeing [33,34,35]. Thus, we can affirm that practically all teenagers in our context experienced an improvement of their quality of life thanks to the acquisition of healthy habits which provided them with a direct decrease in their back pain.

## 5. Conclusions

After participating in the PT activities, the participants learnt how to identify ergonomically incorrect postures. For many students, the increased awareness of the posture adopted in daily life has represented a first step for a change in postural habits.

The students referred to having experienced an improvement in their health status and overall wellbeing, since most participants expressed a total elimination or a significant decrease in their back pain. PT activities: PT activities have been a useful tool for back pain management.

Programs on back education can be organized for large population groups and this does not entail a high financial cost, as no expensive technological material is required, considering that everything that is needed can be found in the environment where the student’s daily life takes place. These programs offer a concrete, effective, and low-cost solution to back pain with other associated benefits, as reported by the participants. The authors of this study propose that these types of PT activities should be introduced as part of the academic curriculum of secondary school within the Spanish Educational system. 

### Limitations and Future Line of Actions

Qualitative research assumes the existence of multiple realities which comprise multiple constructs. Therefore, it is important to always consider the specific study context when reproducing the results of qualitative studies. Further research could measure the flexibility and relationships between the contractions of lumbar and abdominal muscles and, in addition, seek to develop physiotherapy activities in educational centers in other areas of Madrid or other communities in order to create common strategies that benefit the students of different educational centers.

## Figures and Tables

**Table 1:** Information on the duration of interviews and discussion groups.

Data Collection Tools	Mean Time (min)	Total No. of Tools Gathered	Total Time (min)
Group discussion	60	5	300
Interviews	30	119	3570
Total	90	124	3870

**Table 2:** Characteristics of the sample.

Participant	Number	Male	Female	Age	Public School	Private School
Student	49	29	20	15–17 years old	31	18
Teacher	9	2	7	40–60 years old	5	4
Relative	11	3	8	40–50 years old	8	3

**Table 3:** Categories and subcategories of the benefits of physiotherapy activities.

Categories	No. Subcategories	Proportion Subcategories	Quotes Discussion Group	Interview Quotes	Reflexive Diary Quotes	Field Notes
New knowledge acquired	9	22%	1	202	129	37
Improvements experienced by participants	16	40%	4	293	125	26
Daily applicability of physiotherapy activities	15	38%	10	225	122	7
